# Implementing the EffTox dose-finding design in the Matchpoint trial

**DOI:** 10.1186/s12874-017-0381-x

**Published:** 2017-07-20

**Authors:** Kristian Brock, Lucinda Billingham, Mhairi Copland, Shamyla Siddique, Mirjana Sirovica, Christina Yap

**Affiliations:** 1 Cancer Research UK Clinical Trials Unit, Institute of Cancer & Genomic Sciences, University of Birmingham, Birmingham, B15 2TT UK; 2 Paul O’Gorman Leukaemia Research Centre, University of Glasgow, Glasgow, UK

**Keywords:** EffTox, Phase I/II, Dose-finding, Efficacy, Toxicity, CML

## Abstract

**Background:**

The Matchpoint trial aims to identify the optimal dose of ponatinib to give with conventional chemotherapy consisting of fludarabine, cytarabine and idarubicin to chronic myeloid leukaemia patients in blastic transformation phase. The dose should be both tolerable and efficacious. This paper describes our experience implementing EffTox in the Matchpoint trial.

**Methods:**

EffTox is a Bayesian adaptive dose-finding trial design that jointly scrutinises binary efficacy and toxicity outcomes. We describe a nomenclature for succinctly describing outcomes in phase I/II dose-finding trials. We use *dose-transition pathways*, where doses are calculated for each feasible set of outcomes in future cohorts. We introduce the phenomenon of *dose ambivalence*, where EffTox can recommend different doses after observing the same outcomes. We also describe our experiences with *outcome ambiguity*, where the categorical evaluation of some primary outcomes is temporarily delayed.

**Results:**

We arrived at an EffTox parameterisation that is simulated to perform well over a range of scenarios. In scenarios where dose ambivalence manifested, we were guided by the dose-transition pathways. This technique facilitates planning, and also helped us overcome short-term outcome ambiguity.

**Conclusions:**

EffTox is an efficient and powerful design, but not without its challenges. Joint phase I/II clinical trial designs will likely become increasingly important in coming years as we further investigate non-cytotoxic treatments and streamline the drug approval process. We hope this account of the problems we faced and the solutions we used will help others implement this dose-finding clinical trial design.

**Trial registration:**

Matchpoint was added to the European Clinical Trials Database (https://www.clinicaltrialsregister.eu/ctr-search/trial/2012-005629-65/GB) on 2013-12-30.

## Background

The introduction of BCR-ABL tyrosine kinase inhibitors (TKIs; imatinib, dasatinib, nilotinib, bosutinib and ponatinib) has revolutionised the treatment of chronic myeloid leukaemia (CML). The great majority of patients with chronic phase (CP)-CML obtain a durable complete cytogenetic response and the rate of progression to blast phase (BP) is 1 to 2% per annum in the first few years after diagnosis, falling sharply when major molecular response is obtained [1–3]. A minority of patients (<10%) present with *de novo* BP-CML and of these two-thirds are myeloid and one-third lymphoid BP [4]. Despite the use of TKIs, median survival after the diagnosis of BP-CML is between 6.5 and 11 months [5–8], with the majority of long-term survivors being recipients of allogeneic stem cell transplant in second CP [9]. This poor survival is often due to patients developing new mutations, most frequently within the BCR-ABL kinase domain, resulting in resistance to TKIs and further rapid disease progression [10]. Therefore, novel therapies to improve and prolong therapeutic responses in BP-CML are urgently sought.

In the Matchpoint trial, we plan to simultaneously assess co-primary safety and efficacy outcomes for the combination of a novel TKI, ponatinib, with conventional fludarabine, cytarabine and idarubicin (FLAG-IDA) chemotherapy. We believe this to be the first such study in blastic phase CML. It is envisaged that the data will be the first step to improve the treatment of this difficult clinical problem.

The goal of this trial is to find the *optimal* dose of ponatinib to combine with standard doses of FLAG-IDA, rather than merely the maximum tolerable dose. In the so-called *cytostatic* setting, dosing decisions should be guided by patients’ outcomes with regard to efficacy and toxicity, yielding designs for joint phase I/II trials. Different statistical designs define dose optimality in different ways. The method chosen in Matchpoint is described below.

Published clinical trial designs in this arena include extensions of the continual reassessment method [11] (CRM). Braun’s bivariate CRM [12] models separate toxicity and disease progression events. Zhang et al.’s variant of CRM [13] called TriCRM uses an ordered trinary outcome that incorporates response and toxicity. More recently, Wages & Tait [14] introduced a method that uses a latent CRM model to monitor toxicity and selects amongst candidate efficacy models using Bayes factors. Amongst non-CRM alternatives, Wang & Day [15] detailed a utility-maximising approach that assumes responses and toxicity occur in patients according to log-normally distributed patient thresholds.

Thall & Cook [16] introduced EffTox, a Bayesian adaptive dose-finding design that models correlated binary efficacy and toxicity outcomes. A search of PubMed on 17th October 2016 for articles that have cited Thall & Cook [16] returned 54 items. Of these, 36 were methodological in nature, detailing extensions or alternative designs. A further 14 were review articles. Only four articles pertained to the design or reporting of a specific clinical trial. Three of these used the EffTox design [17–19]. The first author is based at the MD Anderson Cancer Center for two of these papers [17, 18], and at the University of Washington for the third [19]. The fourth trial article [20] cites the EffTox paper but uses a randomised trial design. It is not our intention to give a full systematic review but this scoping search suggests that EffTox is not widely used, and scarcely used at all outside the USA. Thall himself admitted that “[Bayesian models for early phase clinical trials] have seen limited use in clinical practice” [21]. In describing our experience using this important dose-finding clinical trial design, we hope to encourage others to use it too. Our proposed solutions to the problems we encountered will expedite the trial design process.

## Methods

### The EffTox design

Thall & Cook [16] introduced the adaptive Bayesian design *EffTox* to facilitate seamless phase I/II dose-finding. EffTox software is published by the MD Anderson Cancer Center at https://biostatistics.mdanderson.org/SoftwareDownload/. We use version 4.0.12.

EffTox uses logit models for the marginal probabilities of efficacy and toxicity at each dose and utility contours (n.b. the EffTox authors use the term *desirability*) to measure the attractiveness of each dose based on the posterior probabilities of efficacy and toxicity.

Let ***y***=(*y*_1_,…,*y*_*n*_) be the *n* doses under investigation. Thall & Cook use the codified doses ***x***=(*x*_1_,…,*x*_*n*_) such that

1 $$  x_{i} = \log{y_{i}} - \sum_{j=1}^{n} \frac{\log{y_{j}}}{n}  $$

For example, a trial of 4 doses, 10 mg, 20 mg, 30 mg and 50 mg, would have ***y***=(10,20,30,50), and ***x***=(−0.85,−0.16,0.25,0.76).

The marginal probabilities of efficacy and toxicity at dose *x* are given by

2 $$  \text{logit}{\pi_{E}(x, \theta)} = \mu_{E} + \beta_{E, 1} x + \beta_{E, 2} x^{2}  $$

and

3 $$  \text{logit}{{\pi}_{T}(x, \theta)} = \mu_{T} + \beta_{T} x  $$

When *β*_*T*_>0, the toxicity probabilities increase monotonically in dose. In contrast, the efficacy curve is not necessarily monotonically increasing. The presence of *β*_*E*,2_ allows for non-linearity and a turning point.

The combined probability model is

4 $$\begin{array}{*{20}l} {\pi}_{a,b} &= ({\pi}_{E})^{a} (1-{\pi}_{E})^{1-a} ({\pi}_{T})^{b} (1-{\pi}_{T})^{1-b} \\ &\quad+ (-1)^{a+b} ({\pi}_{E}) (1-{\pi}_{E}) ({\pi}_{T}) (1-{\pi}_{T}) \frac{e^{\psi}-1}{e^{\psi}+1} \end{array} $$

where *ψ* is an association parameter and (*x*,*θ*)-notation has been suppressed in each function for brevity. Here, *a*,*b* are binary patient-specific variables that denote whether efficacy and toxicity events occurred. For a given patient, *a*=1 means the patient experienced efficacy and *b*=1 means they experienced toxicity.

The EffTox design requires several pieces of information to be elicited from the investigators. Firstly, the statistician must elicit the prior probability of efficacy and toxicity at each dose. Let us label the vector of efficacy probabilities *η*_*E*_, and the toxicity analogue *η*_*T*_. The EffTox software will take these prior beliefs and a desired *effective sample size* (ESS) and convert them into univariate normal priors on each component of ***θ***=(*μ*_*T*_,*β*_*T*_,*μ*_*E*_,*β*_*E*,1_,*β*_*E*,2_,*ψ*). Thall et al. [22] detail the algorithm and advise that ESS should be between 0.5 and 1.5. High values for ESS reflect stronger prior information. The preference is for priors that are strong enough to sensibly guide early dosing decisions but weak enough to be overridden by patient outcomes where they diverge from prior beliefs.

Secondly, the trialists must select parameters to calculate the utility contours [16, 23]. The points $\left ({\pi }_{1,E}^{*}, 0\right)$, $\left (1, {\pi }_{2,T}^{*}\right)$ and $\left ({\pi }_{3,E}^{*}, {\pi }_{3,T}^{*}\right)$ are selected such that they are of equal utility. The quantity ${\pi }_{1,E}^{*}$ is the minimum required probability of efficacy when toxicity is impossible. The quantity ${\pi }_{2,T}^{*}$ is the maximum permissible probability of toxicity when efficacy is guaranteed. The point $\left ({\pi }_{3,E}^{*}, {\pi }_{3,T}^{*}\right)$ is chosen in the first quadrant (i.e. not lying on the *x*- or *y*-axis), representing a point with equal attractiveness to the two other points. EffTox originally used inverse quadratic functions to fit these points but after observing some undesirable behaviour, the authors later advocated using *L* ^*p*^ norms [24]. An *L* ^*p*^ norm is a mathematical tool for generally measuring the distance between two points. The best known is *L* ^2^, the Euclidean norm, that measures the length of a hypotenuse *c* in a right triangle to satisfy *c* ^2^=*a* ^2^+*b* ^2^, where *a* and *b* are the lengths of the other two sides.

Thall et al. [22] stressed the importance of using contours that are steep enough to encourage the design to accept slightly higher probabilities of toxicity when they are compensated with materially higher probabilities of efficacy. This point was developed in detail in Yuan et al. [25]. When the contours are too flat, pathological behaviour can manifest where the design becomes stuck at a sub-optimal dose. This point was unfortunately missed in earlier publications on EffTox [16, 23]. Furthermore, the illustrative example in the original EffTox paper [16] inadvertently uses a family of contours that exhibit pathological behaviour. In order to achieve a design with good properties, Thall advocates selecting three equivalent points that yield a reasonably steep contour, and not trying to elicit points of equal utility from clinicians (please refer to reviewer comments). Fundamentally, trialists should note that EffTox has evolved since its original 2004 publication [16].

The utility of a dose with associated posterior efficacy and toxicity probabilities *π*_*E*_ and *π*_*T*_ is

5 $$  u({\pi}_{E}, {\pi}_{T}) = 1 - \left(\left(\frac{1-{\pi}_{E}}{1-{\pi}_{1,E}^{*}}\right)^{p} + \left(\frac{{\pi}_{T}}{{\pi}_{2,T}^{*}}\right)^{p} \right)^{\frac{1}{p}}  $$

In (5), *p* determines the extent of the curvature of the utility contours. For *p*>1, the contours are convex and for *p*=1, the contours are simply straight lines [24]. The value for *p* is calculated by the EffTox software so that the neutral utility curve intersects $\left ({\pi }_{1,E}^{*}, 0\right)$, $\left (1, {\pi }_{2,T}^{*}\right)$ and $\left ({\pi }_{3,E}^{*}, {\pi }_{3,T}^{*}\right)$.

EffTox uses decision criteria to determine the set of admissible doses based on posterior beliefs. Given trial data for *j* patients, $\mathcal {D} = \left \{ (x_{1}, a_{1}, b_{1}), \ldots, (x_{j}, a_{j}, b_{j})\right \}$, dose *x* is admissible if

6 $$  \text{Pr}\left\{{\pi}_{E}(x, \boldsymbol{\theta}) > \underline{{\pi}}_{E} | \mathcal{D} \right\} > p_{E}  $$

and

7 $$  \text{Pr}\left\{{\pi}_{T}(x, \boldsymbol{\theta}) < \overline{{\pi}}_{T} | \mathcal{D} \right\} > p_{T}  $$

In order to resolve (6) and (7), a prior-to-posterior analysis must be carried out to combine the investigators’ priors with $\mathcal {D}$. This involves solving a six-dimensional integral. The details are given in Thall et al. [16].

The investigators provide values for $\underline {{\pi }}_{E}$, $\overline {{\pi }}_{T}$, *p*_*E*_ and *p*_*T*_ in (6) and (7). The set of doses that are admissible is said to be the admissible set. When a dose selection decision is required (e.g. at the end of a cohort), the admissible set is recalculated. If no dose is admissible, the trial stops and no dose is selected for further research. This may occur if all of the doses are too toxic or insufficiently efficacious, or both. If the admissible set is non-empty, the dose with maximal utility, subject to rules about not skipping untested doses, is recommended to be given to the next cohort or patient.

This iterative process is repeated until the maximum sample size or some pre-defined stopping criteria is reached. The dose recommended after all patients have been treated and evaluated is the dose selected for further research in a later phase trial.

### EffTox in the Matchpoint trial

We chose to use a seamless phase I/II dose-finding design in Matchpoint because we wanted the efficacy events observed to influence the doses selected. We chose to use EffTox because of the readily-available MD Anderson software with which to conduct the trial. Critically, the software performs simulation studies, allowing trialists to hone parameter choices. A summary of our parameter choices appears in Table 1. These are discussed further below.

**Table 1:** EffTox parameters chosen in the Matchpoint trial. These are discussed in the main text

Notation	Interpretation	Value
*N*	Total number of patients	30
*m*	Cohort size	3
*p*_*E*_	Certainty required to infer dose is threshold efficable	0.03
*p*_*T*_	Certainty required to infer dose is threshold tolerable	0.05
$\underline {{\pi }}_{E}$	Minimum efficacy threshold	0.45
$\overline {{\pi }}_{T}$	Maximum toxicity threshold	0.4
${\pi }_{1, E}^{*}$	Required efficacy probability if toxicity is impossible	0.40
${\pi }_{2, T}^{*}$	Permissible toxicity probability if efficacy guaranteed	0.70

In Matchpoint, the binary efficacy event is achieved when patients experience at least a minor cytogenetic response (i.e. <65% Philadelphia chromosome-positive cells), or haematological response (platelets >50×10^9^/L, neutrophils >1.0×10^9^/L and blasts < 5% in the peripheral blood and bone marrow). The binary toxicity outcome is defined by the occurrence of a range of pre-specified adverse events, including any grade 3 or 4 clinically significant non-haematological adverse event, related to ponatinib, that cannot be managed with optimal medical care and likely to endanger the life of the patient or result in long term effects. Both co-primary outcomes are assessed over the eight week period following the commencement of the first cycle of treatment. The first cycle lasts for 28 to 56 days, depending on how long it takes for blood counts to recover.

Of practical importance when using a seamless phase I/II design is that the co-primary outcomes can be assessed over a similar time horizon. It was felt that responses to treatment could be expected after just one cycle and that avoiding dose-limiting toxicity would be instrumental to that. In a scenario where outcomes are assessed over materially different horizons, the trial would proceed at the speed determined by the outcome with the longest assessment period and there would be an increased risk of incomplete data, when one outcome is assessed and the other is waiting to be assessed. This problem was ameliorated with the introduction of the Late Onset EffTox design [26] but we do not use that design here.

We investigate four doses of ponatinib: 15 mg every second day, 15 mg daily, 30 mg daily and 45 mg daily, referenced as dose-levels 1, 2, 3, 4 respectively, as shown in Table 2. For a tractable analysis, we use ***y***=(7.5,15,30,45), and thus ***x***=(−0.97,−0.27,0.42,0.82).

**Table 2:** Doses under investigation in Matchpoint and the investigators’ prior beliefs on rates of efficacy and toxicity

Dose-level	Daily ponatinib dose (mg)	Prior Pr(Eff), *η*_*E*_	Prior Pr(Tox), *η*_*T*_
1	7.5	0.2	0.025
2	15	0.3	0.05
3 (start dose)	30	0.5	0.1
4	45	0.6	0.25

Note, the ponatinib dose labelled 7.5mg per day is actually 15mg every other day

Generally, the clinicians were comfortable providing their prior beliefs on the probability of efficacy and toxicity. They believed a-priori that all doses would be tolerable. There was some debate about the extent to which the probability of efficacy would improve when moving from the third to the highest dose. On balance, it was felt that efficacy would be low at the lowest doses, increase with dose throughout but begin to level-off at the highest dose. This yielded the priors shown in Table 2.

The clinicians were also comfortable specifying $\underline {{\pi }}_{E}$ and $\overline {{\pi }}_{T}$. Conventional chemotherapy regimens like FLAG-IDA can induce complete cytogenetic responses in 20–40% of patients who have progressed to blastic phase [27]. Cortes et al. [28] gave 45 mg of ponatinib daily as a monotherapy to CML patients and observed major cytogenetic response in 23% of 62 patients in blast transformation phase. They also observed very good response rates in chronic phase patients. By combining the treatments, we hope to observe a response rate in excess of 45% so we used $\underline {{\pi }}_{E} = 0.45$. It was our prior belief that only the highest two doses would exceed the minimum efficacy threshold. To achieve this level of efficacy, it was felt that a toxicity rate up to 40% would be acceptable thus we set $\overline {{\pi }}_{T} = 0.40$.

The first cohort will receive dose-level 3 (30 mg) because this is the lowest dose believed a-priori to be sufficiently active. Compared to regular phase I trials, it may seem aggressive to start a dose-finding trial at the third of four doses. A typical phase I design would generally start at a low dose and increase incrementally. Notably, there are simultaneous objectives in dose-finding trials to avoid both *under-* and *over-dosing* patients. Blastic transformation CML is an acute disease phase so there is a strong motivation to avoid sub-therapeutic doses. The tolerability of 45 mg as monotherapy [28] led the clinicians to believe that 30 mg would be tolerable in combination. From here, there is scope to escalate or de-escalate dose as the outcomes dictate.

The values of *p*_*E*_ and *p*_*T*_ in (6) and (7) determine the posterior confidence required to admit the doses as worthy of investigation. Typically, low values are chosen so that even relatively weak beliefs will render doses worthy of investigation in this early phase clinical trial. For instance, Thall et al. [22] use *p*_*E*_=*p*_*T*_=0.1. We initially investigated using the values *p*_*E*_=*p*_*T*_=0.05 but later altered this to *p*_*E*_=0.03. The process of refining these values is described in a later section [29, 30].

**Fig. 1:**
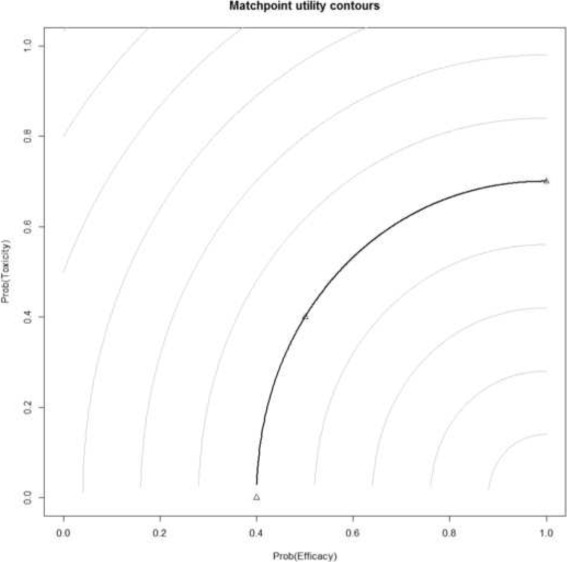
Utility contours in the Matchpoint trial. The neutral utility contour in *bold* joins the points (0.4, 0), (0.5, 0.4) to (1.0, 0.7), represented by *blue triangles*

Rather than specify $\left ({\pi }_{1,E}^{*}, 0\right)$ and $\left (1, {\pi }_{2,T}^{*}\right)$, we agreed three points in the general efficacy-toxicity space (i.e. not on the axes) $\left ({\pi }_{3,E}^{*}, {\pi }_{3,T}^{*}\right)$, $\left ({\pi }_{4,E}^{*}, {\pi }_{4,T}^{*}\right)$ and $\left ({\pi }_{5,E}^{*}, {\pi }_{5,T}^{*}\right)$ such that the points had approximately equal utility. We used the multi-variate solver multiroot in the R [29] package rootSolve [30] to solve the simultaneous equations $u\left ({\pi }_{3,E}^{*}, {\pi }_{3,T}^{*}\right) = u\left ({\pi }_{4,E}^{*}, {\pi }_{4,T}^{*}\right) = u\left ({\pi }_{5,E}^{*}, {\pi }_{5,T}^{*}\right) = 0$. This yielded a neutral utility curve that intersected (39.6%, 0%) and (100%, 67.9%). We rounded to take ${\pi }_{1,E}^{*} = 0.40$ and ${\pi }_{2,T}^{*} = 0.70$, yielding *p*=2.07. Our family of contours are illustrated in Fig. 1. The contours are quite steep where efficacy probabilities are less than 70%. We consider alternative contours in the discussion.

Finally, the value of ESS was chosen by trial-and-error. Thall et al. [22] advise a value in the range (0.5, 1.5). If ESS is too low, the prior can lead to pathological behaviour of the design. Increasing ESS generally improves performance in scenarios that broadly agree with the prior beliefs, and vice-versa. Statisticians and investigators should, however, be mindful of the necessity for the data to override the prior in the event that the priors are wrong. This advocates exercising caution when using inflated ESS values. We arrived at ESS=1.3 because it yielded attractive simulated operating characteristics and sensible dose transitions, as described in the following sections.

### Nomenclature for describing outcomes in phase I/II trials

To expedite the discussion of phase I/II clinical trial conduct, we introduce our nomenclature. Each patient may experience one of four specific outcomes: efficacy without toxicity (E); toxicity without efficacy (T); both (B); or neither (N). Let us string these symbols behind a numerical dose-level to denote the outcomes of cohorts of patients. For instance, 2EET denotes a cohort of three patients that were given dose-level 2, two of whom experienced efficacy only and one who experienced toxicity. These strings can be concatenated to describe the outcomes of several cohorts consecutively. For example the path 2EET 3EBB extends our previous scenario. After the first cohort, the trial escalated to dose-level 3. The next cohort of three were treated at this dose and all three patients experienced efficacy. Unfortunately, two of them also experienced toxicities. Using our notation, this information is unambiguously conveyed in 8 characters.

In phase I/II, it is inadvisable to reduce patients’ outcomes to simple tallies of efficacy and toxicity events because of the complication that patients may experience both events or neither. For instance, the design may recommend a different dose after observing NTE than it would after observing NNB, even though both cohorts contain a single efficacy event and a single toxicity event. In the first example, the events are experienced by different patients whereas in the latter, they are experienced by the same patient. The distinction is especially pertinent in EffTox because the *ψ* parameter models the association between efficacy and toxicity.

The described notation combines simple codification of dose-levels and patient outcomes to succinctly and unambiguously describe pathways through phase I/II dose-finding trials. We use it in the next section to define dose transition pathways, and in following sections to discuss the potential problems of outcome ambiguity and dose ambivalence, and to aid trial planning.

### Dose transition pathways

We found it greatly beneficial to prospectively analyse how our dose-finding design would behave with respect to cohorts by supposing each feasible set of future patient outcomes and calculating the model advice in each. From a given starting point, we look to identify the conditions under which the design would escalate dose, stay at a dose, de-escalate dose, or recommend that the trial stops.

The principle of analysing *dose-transition pathways* (DTPs) was introduced in a dose-finding trial [31] and has been submitted for publication by Yap, et al. The example in Table 3 shows the complete set of DTPs for cohort 2 having observed 3TTT in cohort 1 and de-escalating to dose-level 2.

**Table 3:** After observing 3TTT in cohort 1, cohort 2 is recommended to receive dose-level 2

Cohort 2 outcomes	Dose for cohort 3
2NNN	3
2NNE	1
2NNT	Stop trial
2NNB	1
2NEE	1
2NET	1
2NEB	1
2NTT	Stop trial
2NTB	1
2NBB	1
2EEE	1
2EET	1
2EEB	1
2ETT	1
2ETB	1
2EBB	1
2TTT	Stop trial
2TTB	1
2TBB	1
2BBB	1

The dose recommended for cohort 3 depends on the outcomes in cohort 2, as depicted by these dose transition pathways

At dose-selection meetings in the Matchpoint trial, we make frequent use of DTPs. We are particularly interested to learn the outcomes that would have to manifest to change dose or stop the trial.

Dose decisions by EffTox, as projected in advance by DTPs, can sometimes seem counter-intuitive. This may be because the admissibility criteria and utility contours work in unison to: a) filter out doses believed to be too toxic or insufficiently active; and b) identify the most desirable of the admissible doses. This particularly could be the case relatively early in the trial stages when only a small amount of discrete data is available.

Table 3 shows DTPs for a single future cohort but that need not be a constraint. We use DTPs in Matchpoint to analyse every feasible outcome of the next few cohorts. DTPs can be calculated for several subsequent cohorts, or even an entire trial. However, the number of possible paths grows geometrically with the number of cohorts being considered. Each evaluable patient will experience exactly one of E, T, N or B, independent of the other patients. With cohorts of three, the number of distinct outcomes for a single cohort is 20, as shown in Table 3, hence the number of feasible DTPs for the next two and three cohorts are 20^2^ and 20^3^ respectively. Thus, the limitation of what can be depicted on printed pages tends to limit our DTP analysis to no more than the next two cohorts of three patients.

The *DTPs* tutorial in the clintrials package can be used to reproduce Table 3. Refer to the *Availability of data and software* section at the end of this article.

### Posterior utility

Thall & Cook work with utility as a function of the mean posterior efficacy and toxicity probabilities of the doses. In contrast, we consider the posterior distribution of utility scores. For example, the posterior mean utility of dose *x* is

8 $$  \hat{u}({\pi}_{E}, {\pi}_{T}) | \mathcal{D}) = \frac{\int u({\pi}_{E}, {\pi}_{T}) \mathcal{L}(\boldsymbol{\theta} | \mathcal{D}) f(\boldsymbol{\theta}) d\boldsymbol{\theta}}{\int \mathcal{L}(\boldsymbol{\theta} | \mathcal{D}) f(\boldsymbol{\theta}) d\boldsymbol{\theta}}  $$

where $\mathcal {L}$ is the likelihood function given in EffTox [16], *f*(***θ***) is the parameter prior distribution, and *π*_*E*_ and *π*_*T*_ are shorthand for *π*_*E*_(*x*,***θ***) and *π*_*T*_(*x*,***θ***) respectively.

**Fig. 2:**
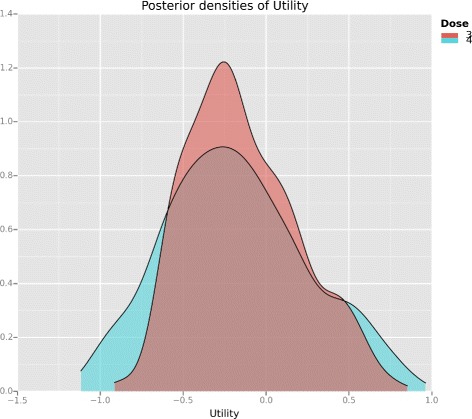
Posterior utility after 3 patients. Posterior densities of the utility of doses 3 and 4. After three patients with outcomes 3NTE, the densities largely occupy the same space and dose ambivalence is likely

**Fig. 3:**
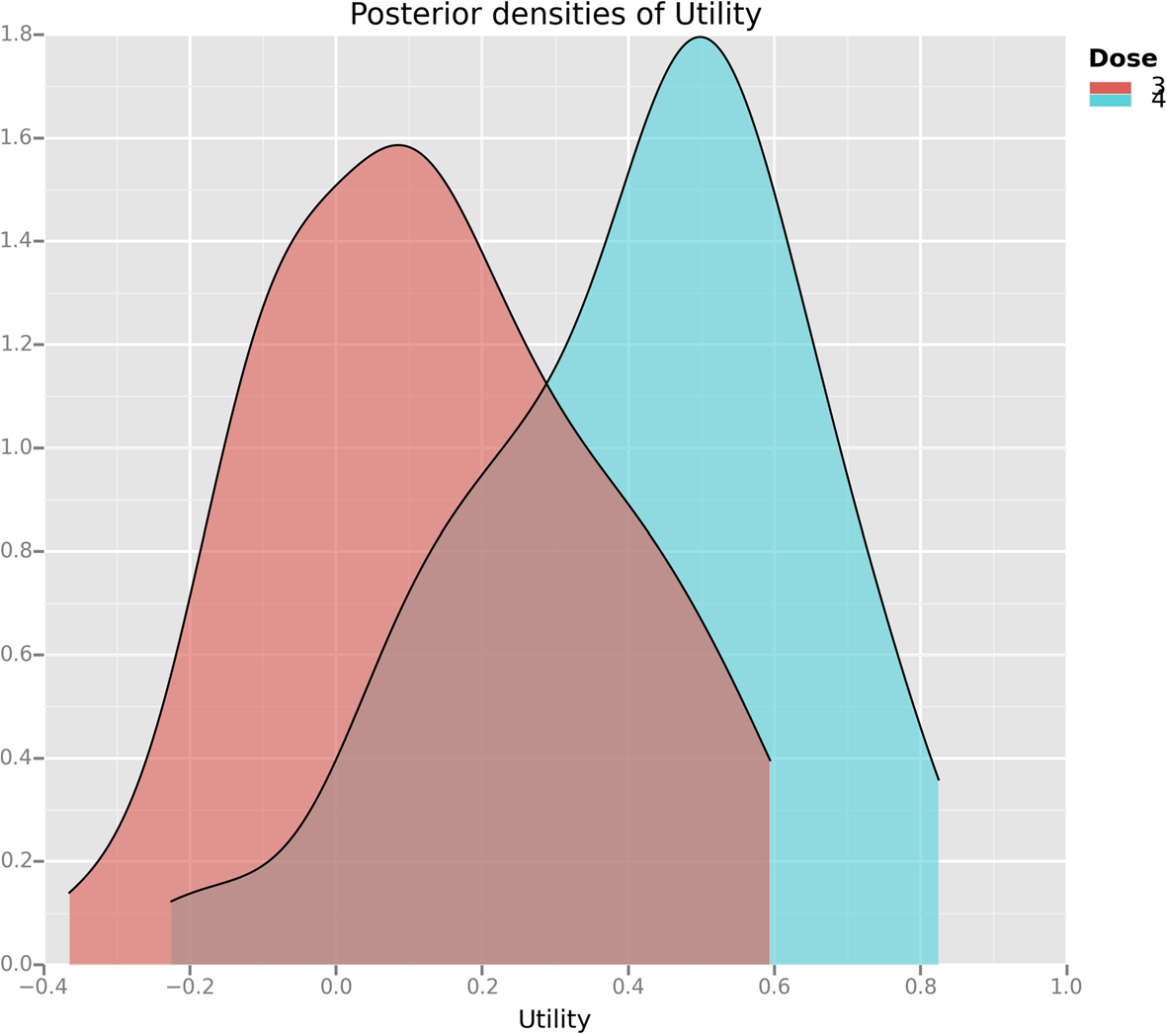
Posterior utility after 15 patients. Posterior densities of the utility of doses 3 and 4. In contrast to Fig. 2, after 15 patients with outcomes 2NNN 3ENN 4EBE 3TEE 4NEE, the posterior utilities are quite distinct and dose ambivalence is much less likely

The *Utility* tutorial in the clintrials package shows how to calculate posterior plots of utility like those in Figs. 2 and 3. Refer to the *Availability of data and software* section at the end of this article.

## Results

In this section we describe some problems we encountered and the practical solutions we implemented using the methods described in the previous section. All data in this section are hypothetical or simulated. No actual trial outcomes are presented.

### Outcome ambiguity

Patients in the blastic transformation phase of CML under study in the Matchpoint trial are particularly sick. The FLAG-IDA regimen is toxic and the addition of ponatinib only increases the potential for adverse events. Periodic dose-selection meetings are a feature of dose-finding studies, where early safety and efficacy outcomes are reviewed and a new dose for the next patient or cohort is selected. Sometimes, because of the frail nature of the patients, efficacy assessments are temporarily delayed. This *outcome ambiguity* presents a challenge for dose-selection because the decision seemingly requires that full patient outcomes be available. However, we have already seen that this is not necessarily the case. In the scenario depicted in Table 3, we know that at least one E or B event in cohort 2 is enough to know with certainty that the trial will proceed to cohort 3 using dose-level 1. If one of the patients experiences E or B in cohort 2, the dose-decision is independent of the other two patients so it does not matter, purely from a dose-decision perspective, if some of the outcome information is temporarily missing for the other patients in cohort 2.

Naturally, this phenomenon does not always occur and there are many occasions when every patient’s outcomes will be required promptly to know the course of action in the subsequent cohort. Furthermore, it is important that outcomes for cohort 2 are finalised before trying to establish doses for cohorts after cohort 3, for example, because all patient outcomes affect the dosing decision in model-based dose-finding designs. The described method merely offers short-term respite in *some* occasions if a small number of data-points are *temporarily* missing.

The general problem of partially observed outcomes has been addressed more formally by Jin et al. [26] with their Late Onset EffTox (LO-ET) design. This method incorporates time-to-event data and uses the predictive posterior distribution of treated yet non-assessed patients to speed up the trial.

### Dose ambivalence

The probability model uses six parameters for which prior distributions are stipulated. After patient outcomes are observed, posterior estimates of efficacy and toxicity come from evaluating a six-dimensional integral, one dimension for each parameter. Such integrals are approximated numerically rather than solved analytically and this leads to estimation error. Typically, early phase clinical trials do not use a large number of patients so the amount of information in the trial will usually be quite low, i.e. the number of patients divided by the number of parameters being estimated will be lower than a typical phase II or III trial. The combined effect of these two sources of variability is that the outputs of the EffTox model are subject to quite a lot of uncertainty, especially in early cohorts.

This uncertainty is a feature of all sequentially adaptive clinical trial designs that must rely on small to moderate sample sizes. For instance, Thall et al. [32] demonstrate in their Fig. 2 the uncertainty present in posterior utility estimates of two-agent dose combinations after *n*=60 patients.

An occasional unwelcome consequence of this uncertainty in EffTox is that the model can occasionally make different dose recommendations based on the same patient outcomes. This is obviously undesirable in a clinical trial where a categorical course of action is sought. In Matchpoint, we referred to this phenomenon as *dose ambivalence*. We noticed this whilst analysing DTPs.

The posterior utility density curve in Fig. 2 demonstrates the difficulty a utility-maximising design like EffTox faces when few patient outcomes have been observed and two doses have very similar utility scores. It shows the posterior beliefs on the utilities of dose-levels 3 and 4 after observing 3NTE in cohort 1. For clarity in illustration, dose-levels 1 and 2 are not shown. The posterior curves are estimated using Monte Carlo integration, bootstrap sampling and density smoothing via the *ggplot* function in R [29]. Figure 2 shows that the distribution for dose 4 has slightly greater variability but that the two utilities have approximately equal mean and mode. When the posterior utilities for the two doses are so similar, it is difficult for the design to reliably choose between them and dose ambivalence is the likely result. After observing outcomes 3NTE in the first cohort, the design sometimes recommends dose-level 3 for the next cohort, and sometimes dose-level 4. This ambiguity manifests because the two doses are both admissible, have similar utility scores, and the Bayesian update integral is imperfectly calculated. This happens when using the MD Anderson implementation of the EffTox software that uses the spherical radial method of Monahan and Genz [33] to estimate the posterior integrals, and our own implementation in clintrials that uses Monte Carlo integration.

It is possible to calculate the integral more precisely by increasing the number of points in the numerical algorithm but this risks missing an important message. If the dose-recommendation is not consistent when calculated to a reasonable numerical precision, the design is telling us that it is difficult to pick between the doses. It could be that several doses have similar utility scores, as we have seen. Alternatively, it could be that a dose is very close to the boundary for inclusion in the admissible set and its eligibility determines the immediately preferable course.

Figure 3 shows similar curves after 15 patients with outcomes 2NNN 3ENN 4EBE 3TEE 4NEE. In contrast, the posterior utilities are now quite distinct and a consistent dose decision is almost guaranteed.

During dose-update decisions, we ran the dose selection decision multiple times to identify if dose ambivalence was present. When it did manifest, we used posterior utility plots like Figs. 2 and 3 and the view of independent clinicians and statisticians on the Matchpoint steering committee to support decision-making.

The *Ambivalence* tutorial in the clintrials package presents a Matchpoint scenario with dose ambivalence and demonstrates repeated calculation of the dose-decision. Refer to the *Availability of data and software* section at the end of this article.

### Changing *p*_*E*_ to avoid premature stopping

In this section, we describe an unintended flaw in the original parameterisation of our Matchpoint design that lead to premature stopping. This undesirable behaviour was uncovered by analysing DTPs. We advise trialists to prospectively analyse DTPs of the early trial cohorts yielded by candidate designs before starting the trial.

We commenced Matchpoint with both *p*_*E*_ and *p*_*T*_ set to 0.05 so that the design only had to be at least 5% sure that a dose was efficacious and safe to include it in the admissible set. These values are lower than those used conventionally. Thall et al. [22] for example use *p*_*E*_=*p*_*T*_=0.1. However, we had cause to reduce *p*_*E*_ further to 0.03 in Matchpoint. Output from the official EffTox software in Table 4 reveals the cause.

**Table 4:** EffTox posterior beliefs after 3TTT

	Dose 1	Dose 2	Dose 3	Dose 4
Utility	-0.489	-0.534	-0.777	-0.817
Pr(${\pi }_{E} > \underline {{\pi }}_{E}$)	0.079	0.037	0.060	0.200
Pr(${\pi }_{T} < \overline {{\pi }}_{T}$)	0.919	0.758	0.051	0.005
Admissible under *p*_*E*_=0.05, *p*_*T*_=0.05	1	0	1	0
Admissible under *p*_*E*_=0.03, *p*_*T*_=0.05	1	1	1	0

The values for *p*_*E*_ and *p*_*T*_ determine the admissible doses

Having observed 3TTT in the first cohort, all doses are believed to be unattractive. The most attractive dose is actually dose-level 1, so the design would like to de-escalate. However, the design cannot go straight to dose-level 1 because the restriction to not skip untried doses requires that dose-level 2 is tested first. The software does not allow this feature to be turned off. However, with *p*_*E*_=0.05, dose-level 2 is actually inadmissible so the design cannot de-escalate.

The problem is potentially exacerbated by the fact that Pr(${\pi }_{T} < \overline {\pi }_{T}$) is very close to the value *p*_*T*_=0.05 for dose-level 3. If this probability is estimated to be slightly less than 0.05, as is plausible with just 3 data-points and a six-dimensional Bayesian integral solved numerically, then dose-level 3 becomes inadmissible also. Under these circumstances, with dose-level 4 inadmissible too on account of excess toxicity, the design cannot recommend a dose so it advocates stopping. This occurs in a material percentage of the dose decision invocations using the EffTox software. Using *p*_*E*_=*p*_*t*_=0.1 further exacerbates the problem, stopping categorically after 3TTT and 3NTT.

Observing three toxicities in the first three patients is clearly a grave situation. However, we should be mindful of the play of chance and the extent of our knowledge on the event rates. The lower bound of the 95% confidence interval for a binomial proportion having observed three events in three trials is 29.2% using the exact method, and 43.9% using the Wilson method. Hence, the true toxicity rate could plausibly be much lower than the 100% rate observed with 3TTT. Also, we have no direct knowledge of the toxicity rates at the other dose-levels, only the information extrapolated by our model from the toxicities observed at dose-level 3. We prefer that the design be able to de-escalate after observing 3TTT and the trial continue. We achieved this by reducing *p*_*E*_. We advise fellow trialists to study DTPs routinely, especially in early cohorts, to spot similar behaviour. When doing so, as we have illustrated here, it is important to consider the interaction of the contours and the admissibility criteria [34].

### Operating characteristics

Once a complete set of parameters has been proposed, we learn how the design performs on average by simulation.

Blastic transformation phase CML is relatively rare. It was felt that 30 patients was the upper feasible limit to recruit in a reasonable time frame. We selected the highest feasible sample size to maximise our chances of identifying the optimal dose. Thirty patients would be sufficient to offer the attractive operating characteristics discussed below. We planned to use cohorts of three because this would offer an attractive balance between the frequency of model updates and opportunity for the design to explore the doses.

The EffTox software provides the ability to simulate the outcome of thousands of trials using the chosen design and some assumed true efficacy and toxicity curves. In practice, of course, the true curves are unknown. We choose a variety of scenarios for simulation that will provide pertinent information on the behaviour of the design in real usage.

Trialists should assess performance in a set of clinically relevant scenarios. One of the scenarios should closely resemble the investigators’ prior beliefs, as this represents the anticipated outcome. We would expect the model to perform very well here. The setting for any clinical trial is that we are unsure of the truth so the range of scenarios in which our design performs well should reflect our ignorance. We considered how the design would perform if adverse circumstances prevailed. To these ends, we advocate analysing performance when (i) no doses are tolerable, and (ii) no doses are efficacious. In these scenarios, the desirable behaviour is to stop. As the clinical scenario dictates, we might also advocate analysing scenarios where the true efficacy and toxicity curves are not monotonically increasing.

In Table 5 we analyse in six indicative scenarios the performance of our chosen EffTox model in Matchpoint, labelled ESS=1.3. We have also given the performance of two other models with priors on ***θ*** recalibrated using the EffTox software to give ESS set to 0.5 and 1.5, being the recommended lower and upper limits on ESS advised by Thall et al. [22]. These convey the feasible range of performance, holding all other parameters constant. In every other regard, the three models are exactly the same.

**Table 5:** Operating characteristics of EffTox designs with 30 patients in cohorts of 3 and ESS=0.5, 1.3 and 1.5

Scenario		Dose 1	Dose 2	Dose 3	Dose 4	Stop
	Pr(Eff)	0.20	0.30	0.50	0.60	
1:	Pr(Tox)	0.03	0.05	0.10	0.30	
monotonic,	Utility	-0.33	-0.17	0.16	**0.22**	
dose 4	ESS=0.5	0.01	0.01	0.34	***0.63***	0.01
optimal	ESS=1.3	<0.01	<0.01	0.22	***0.76***	<0.01
	ESS=1.5	<0.01	<0.01	0.22	***0.77***	<0.01
	Pr(Eff)	0.40	0.60	0.75	0.79	
2:	Pr(Tox)	0.10	0.25	0.55	0.60	
monotonic,	Utility	-0.01	**0.25**	0.12	0.08	
dose 2	ESS=0.5	0.06	***0.59***	0.32	<0.01	0.03
optimal	ESS=1.3	0.03	***0.60***	0.35	<0.01	0.01
	ESS=1.5	0.03	***0.57***	0.39	<0.01	0.01
	Pr(Eff)	0.25	0.40	0.60	0.60	
3:	Pr(Tox)	0.10	0.20	0.38	0.42	
eff. plateau,	Utility	-0.26	0.04	**0.15**	0.12	
dose 3	ESS=0.5	0.03	0.10	***0.70***	0.13	0.04
optimal	ESS=1.3	0.01	0.10	***0.73***	0.13	0.02
	ESS=1.5	0.01	0.09	***0.73***	0.15	0.02
	Pr(Eff)	0.50	0.60	0.70	0.80	
4:	Pr(Tox)	0.20	0.20	0.20	0.20	
tox. plateau,	Utility	0.12	0.28	0.43	**0.57**	
dose 4	ESS=0.5	0.02	0.03	0.61	***0.34***	<0.01
optimal	ESS=1.3	<0.01	0.02	0.47	***0.50***	<0.01
	ESS=1.5	<0.01	0.01	0.47	***0.51***	<0.01
	Pr(Eff)	0.05	0.08	0.20	0.25	
	Pr(Tox)	0.05	0.08	0.12	0.14	
5:	Utility	-0.58	-0.54	-0.34	-0.26	
all doses	ESS=0.5	0.06	0.03	0.01	0.37	***0.53***
inactive	ESS=1.3	0.06	0.07	0.02	0.34	***0.51***
	ESS=1.5	0.07	0.08	0.02	0.36	***0.48***
	Pr(Eff)	0.05	0.08	0.12	0.25	
6:	Pr(Tox)	0.60	0.65	0.70	0.80	
all doses	Utility	-0.78	-0.78	-0.76	-0.67	
too toxic	ESS=0.5	0.09	0.01	0.01	0.01	***0.88***
and inactive	ESS=1.3	0.06	0.01	0.01	0.01	***0.91***
	ESS=1.5	0.04	0.01	0.01	0.01	***0.93***

In Matchpoint, we use the model with ESS=1.3. In each scenario section, the probabilities of efficacy and toxicity are given, in addition to the utility scores determined by (5). Dose *i* is the probability that dose-level *i* is recommended for further research, for *i*=1,…,4. Stop is the probability of stopping and recommending no dose. In rows pertaining to design performance, the correct decision is in bold and the admissible decisions are underlined. When stopping is the correct decision, stopping is the only admissible decision. The EffTox software gives selection probabilities to the nearest whole percent

We naturally seek a design that selects the optimal dose most reliably. Our ESS=1.3 design with 30 patients makes the optimal decision with probability at least 50% in each scenario. In scenarios 1, 3 and 6, it makes the optimal choice in the great majority of iterations.

Scenarios 1 and 4 show the benefit of a modestly more informative prior. Through the addition of prior information approximately equivalent to one patient (i.e. increasing the effective sample size of the prior from 0.5 to 1.3 or 1.5), the probability that the design selects the optimal dose is increased by up to 17%. Investigators will naturally ponder the existence of the opposite effect, i.e. an increased propensity to do the wrong thing when the prevailing scenario disagrees with the prior. Scenario 3 shows that this is not necessarily the case. The designs with more informative priors actually perform slightly better, despite a shape of efficacy curve that disagrees with the prior.

Table 6 shows the mean probability that each design variant identifies the optimal decision, given the six scenarios in Table 5. We see that our design is the superior of the three presented. The variant with ESS=0.5 has inferior performance, mostly for the reasons discussed. The variant with ESS=1.5 is only modestly inferior but provides no reason to be preferred to our design.

**Table 6:** Mean probabilities of performing the optimal decision in the scenarios presented in Table 5

Design variant	Mean Pr(Optimal decision)
ESS=0.5	0.612
ESS=1.3	0.668
ESS=1.5	0.65

We investigated by simulation the larger sample size *n*=60. The extra patients greatly improve performance in some scenarios. In scenario 2, the probability of selecting dose 2 increases by 20 to 80%. Similarly, the chances of correctly stopping early in scenario 5 increase by 27 to 78%. In many clinical scenarios, recruiting a higher number of patients is warranted in a phase I-II trial because of the associated improvement in performance and the presence of efficacy assessment that may abrogate a further traditional phase II trial. Phase I-II trials are an opportunity to optimise the delivery of a new agent. In the Matchpoint scenario, unfortunately, higher recruitment was simply not feasible because of the rarity of BP-CML.

We also investigated the impact of using *p*_*E*_=*p*_*T*_=0.1. The chances of stopping in scenario 5 are improved by 30%. As expected, the reciprocal effect is that the design stops slightly more frequently in scenarios like 3 where an optimal dose exists.

As well as their propensity to make the correct decision, we also discriminate designs on how they allocate doses to patients. A design that always makes the correct decision but treats every patient at an over dose would not desirable, or indeed ethical. Table 7 gives the mean number of patients allocated to each dose in the scenarios presented in Table 5.

**Table 7:** Numbers of patients allocated to doses in the six scenarios and three EffTox variants presented in Table 5

Scenario		Dose 1	Dose 2	Dose 3	Dose 4	Sum
1:	Pr(Eff)	0.20	0.30	0.50	0.60	
monotonic,	Pr(Tox)	0.03	0.05	0.10	0.30	
dose 4	Utility	-0.33	-0.17	0.16	**0.22**	
optimal	ESS=0.5	0.7	0.6	12.1	***16.4***	29.8
	ESS=1.3	0.2	0.2	9.8	***19.6***	29.8
	ESS=1.5	0.1	0.1	9.5	***20.1***	29.8
2:	Pr(Eff)	0.40	0.60	0.75	0.79	
monotonic,	Pr(Tox)	0.10	0.25	0.55	0.60	
dose 2	Utility	-0.01	**0.25**	0.12	0.08	
optimal	ESS=0.5	1.5	***11.5***	16.0	0.4	29.4
	ESS=1.3	0.8	***11.6***	16.9	0.6	29.9
	ESS=1.5	0.7	***10.3***	18.2	0.7	29.9
3:	Pr(Eff)	0.25	0.40	0.60	0.60	
eff. plateau,	Pr(Tox)	0.10	0.20	0.38	0.42	
dose 3	Utility	-0.26	-0.04	**0.15**	0.11	
optimal	ESS=0.5	1.1	2.8	***21.8***	3.7	29.4
	ESS=1.3	0.5	2.5	***22.2***	4.4	29.6
	ESS=1.5	0.4	2.0	***22.1***	5.2	29.7
4:	Pr(Eff)	0.50	0.60	0.70	0.80	
tox. plateau,	Pr(Tox)	0.20	0.20	0.20	0.20	
dose 4	Utility	0.12	0.28	0.43	**0.57**	
optimal	ESS=0.5	0.5	1.0	19.3	***9.2***	30.0
	ESS=1.3	0.1	0.7	15.9	***13.3***	30.0
	ESS=1.5	0.1	0.3	15.9	***13.7***	30.0
5:	Pr(Eff)	0.05	0.08	0.20	0.25	
all doses	Pr(Tox)	0.05	0.08	0.12	0.14	
inactive	Utility	-0.58	-0.54	-0.34	-0.26	
	ESS=0.5	2.4	2.4	4.7	14.1	23.6
	ESS=1.3	1.5	1.9	4.7	15.3	23.4
	ESS=1.5	1.4	1.7	4.5	16.0	23.6
6	Pr(Eff)	0.05	0.08	0.12	0.25	
all doses	Pr(Tox)	0.60	0.65	0.70	0.80	
too toxic	Utility	-0.78	-0.78	-0.76	-0.67	
and inactive	ESS=0.5	2.6	3.0	4.0	0.9	10.5
	ESS=1.3	1.1	2.8	5.2	0.8	9.9
	ESS=1.5	1.0	2.8	5.3	0.9	10.0

Dose *i* is the mean number of patients receiving dose-level *i*, for *i*=1,…,4. Row sums are also given in Sum. Sum ≠30 in some instances due to trials stopping early. Patients allocated to the optimal dose are given in bold and underlined for the admissible doses

Of the three designs presented, our chosen design uses the fewest patients in scenarios 5 and 6, where the correct decision is to stop the trial. On the four remaining scenarios, our chosen design allocates the most patients on average to the optimal dose in two scenarios. We see this as further reason to prefer the design with ESS=1.3.

In summary, we have shown by simulation that our selected EffTox parameterisation performs well in six scenarios. We have demonstrated that it stops reliably in situations where all doses are too toxic or inefficacious. We have also shown it to perform well in a scenario that broadly matches our prior, and in scenarios that depart from our prior. Lastly, we have demonstrated that our chosen parameterisation with effective sample size set to 1.3 is superior to alternatives with ESS set to 0.5 and 1.5, in terms of the probability of making the correct decision, and in the allocation of patients to favourable doses.

## Discussion

Finalising an EffTox design is generally an iterative process. The inferences from analysing dose transition pathways and simulations will naturally lead to re-parameterisation and further testing. It is our general preference to first hone the dose transitions. For the reasons described, we pay particular attention to the earliest circumstances under which the trial would stop. The investigators should agree that these circumstances are dire enough to warrant closing the trial. We also look for any sign that the design seems reluctant to select a dose. This could suggest unsuitable priors or inappropriate parameter choices. It should be stressed, however, that EffTox exists to guide our sequential selection of doses based on patient outcomes. The trialists should not stipulate every conceivable dose path and select parameters that replicate their choices. This approach would preclude the use of a model at all. Rather, in our opinion, the parameters should be selected for generally acceptable behaviour, with due consideration given to the extremes.

Once an acceptable parameterisation has been proposed, the performance of the design should be assessed by simulation under a broad range of scenarios. The design should stop sufficiently early and reliably when all doses are too toxic. In scenarios where optimal and/or acceptable doses exist, the design should select those with acceptable probability. Refinements to the parameterisation here will likely require the trial designer to consider how they affect the behaviour of the design in dose transition, and thus the circularity of the challenge is illustrated.

We have considered even steeper contours, as stressed by Thall et al. [22] and Yuan et al. [25]. They did not lead to superior performance in the particular scenarios we have chosen. This is likely due to the fact that our contours are steep for efficacy probabilities as high as 70%, which we consider to be the clinically plausible scenario in BP-CML. However, the point remains that trade-off contours should be steep to motivate the design to accept higher probabilities of efficacy for acceptably higher probabilities of toxicity.

EffTox is a powerful yet underused statistical design for seamless phase I/II dose-finding clinical trials. Model-based designs are becoming more important as trialists and funders move away from so-called “up-and-down” designs [35] like 3+3. This trend will be further driven as investigators research treatments for which the *maximum-tolerated* dose is unlikely to coincide with the most effective dose. We have described the obstacles we faced in implementing EffTox in Matchpoint and our approach to overcoming those obstacles in the hope that this will help trialists.

We were able to choose EffTox because our co-primary outcomes efficacy and toxicity were assessed over a similar time-frame. EffTox and other dose-finding designs with co-primary outcomes would not have been suitable if one had required a longer assessment period.

A key reason for selecting EffTox was the readily available, free software provided by the MD Anderson Cancer Center for performing dose calculations and simulations of trial operating characteristics. With the many time pressures that come with working in an academic clinical trials unit, it was a tremendous advantage to have reliable software with which to design and run this trial. One of the drawbacks of using compiled software was our inability to alter or add certain behaviours. For instance, we might have suppressed the no-skipping in de-escalation rule, had that been possible. The desire to routinely calculate dose-transition pathways led us to develop an open-source implementation of EffTox in clintrials [34].

## Conclusion

Joint phase I/II clinical trials will likely become more common in coming years as we investigate non-cytotoxic treatments and streamline the drug approval process. EffTox is an important trial design because it addresses both of these needs. However, it requires parameterisation and preliminary calculation. Choices for parameters can potentially have both intended and unintended consequences. The process of finalising an EffTox design is inherently iterative. We aim to help trialists implement the design by describing our solutions to the problems we faced.

We have discussed the parameters we chose and how we selected them. We have stressed the need to look at the dose transition pathways, particularly in the early stages when few outcomes are observed, and at the circumstances that would lead to the trial’s termination. We have highlighted the problem of dose ambivalence, illustrated graphically, and suggested a pragmatic solution. We have described the problem of outcome ambiguity, and how dose-transition pathways can mitigate the problem in the short term, allowing the dose-finding trial to proceed whilst clinical evaluation is ongoing. Finally, we have advised on the simulation scenarios that should be considered. We hope this paper will help other investigators implement this important dose-finding clinical trial design.

## Abbreviations

BP: Blast phase
CML: Chronic myeloid leukaemia
CP: Chronic phase
CRM: Continual reassessment method
DLT: Dose-limiting toxicity
MTD: Maximum tolerable dose
SCT: Stem cell transplant
TKI: Tyrosine kinase inhibitor

## References

[1] Druker BJ, Guilhot F, O’Brien SG, Gathmann I, Kantarjian H, Gattermann N, Deininger MWN, Silver RT, Goldman JM, Stone RM, Cervantes F, Hochhaus A, Powell BL, Gabrilove JL, Rousselot P, Reiffers J, Cornelissen JJ, Hughes T, Agis H, Fischer T, Verhoef G, Shepherd J, Saglio G, Gratwohl A, Nielsen JL, Radich JP, Simonsson B, Taylor K, Baccarani M, So C, Letvak L, Larson RA (2006). Five-Year Follow-up of Patients Receiving Imatinib for Chronic Myeloid Leukemia. N Engl J Med.

[2] Jabbour E, Kantarjian HM, Saglio G, Steegmann JL, Shah NP, Boque C, Chuah C, Pavlovsky C, Mayer J, Cortes J, Baccarani M, Kim DW, Bradley-Garelik MB, Mohamed H, Wildgust M, Hochhaus A (2014). Early response with dasatinib or imatinib in chronic myeloid leukemia : 3-year follow-up from a randomized phase 3 trial (DASISION). Blood.

[3] Larson R, Hochhaus A, Hughes T, Clark R, Etienne G, Kim DW, Flinn I, Kurokawa M, Moiraghi B, Yu R, Blakesley R, Gallagher N, Saglio G, Kantarjian H (2012). Nilotinib vs imatinib in patients with newly diagnosed Philadelphia chromosome-positive chronic myeloid leukemia in chronic phase : ENESTnd 3-year follow-up This article has been corrected since Advance Online Publication and a corrigendum is also printed. Leukemia.

[4] Hehlmann R (2012). How I treat CML blast crisis How I treat How I treat CML blast crisis. Blood.

[5] Druker BJ, Sawyers CL, Kantarjian H, Resta DJ, Reese SF, Ford JM, Capdeville R, Talpaz M (2001). Activity of a specific inhibitor of the BCR-ABL tyrosine kinase in the blast crisis of Chronic Myeloid Leukemia and Acute Lymphoblastic Leukemia with the Philadelphia chromosome. N Engl J Med.

[6] Giles FJ, Kantarjian HM, le Coutre PD, Baccarani M, Mahon FX, Blakesley RE, Gallagher NJ, Gillis K, Goldberg SL, Larson RA, Hochhaus A, Ottmann OG (2012). Nilotinib is effective in imatinib-resistant or -intolerant patients with chronic myeloid leukemia in blastic phase,. Leukemia.

[7] Palandri F, Castagnetti F, Testoni N, Luatti S, Marzocchi G, Bassi S, Breccia M, Alimena G, Pungolino E, Rege-Cambrin G, Varaldo R, Miglino M, Specchia G, Zuffa E, Ferrara F, Bocchia M, Saglio G, Pane F, Alberti D, Martinelli G, Baccarani M, Rosti G (2008). Chronic myeloid leukemia in blast crisis treated with imatinib 600 mg: Outcome of the patients alive after a 6-year follow-up. Haematologica.

[8] Saglio G, Hochhaus A, Goh YT, Masszi T, Pasquini R, Maloisel F, Erben P, Cortes J, Paquette R, Bradley-Garelik MB, Zhu C, Dombret H (2010). Dasatinib in imatinib-resistant or imatinib-intolerant chronic myeloid leukemia in blast phase after 2 years of follow-up in a phase 3 study: Efficacy and tolerability of 140 milligrams once daily and 70 milligrams twice daily. Cancer.

[9] Jain N, Van Besien K (2011). Chronic Myelogenous Leukemia: Role of Stem Cell Transplant in the Imatinib Era. Hematol Oncol Clin N Am..

[10] Soverini S, Colarossi S, Gnani A, Rosti G, Castagnetti F, Poerio A, Iacobucci I, Amabile M, Abruzzese E, Orlandi E, Radaelli F, Ciccone F, Tiribelli M, Di Lorenzo R, Caracciolo C, Izzo B, Pane F, Saglio G, Baccarani M, Martinelli G (2006). Contribution of ABL kinase domain mutations to imatinib resistance in different subsets of Philadelphia-positive patients: By the GIMEMA working party on chronic myeloid leukemia. Clin Cancer Res.

[11] O’Quigley J, Pepe M, Fisher L (1990). Continual reassessment method: a practical design for phase 1 clinical trials in cancer. Biometrics.

[12] Braun TM (2002). The bivariate continual reassessment method: Extending the CRM to phase I trials of two competing outcomes. Control Clin Trials.

[13] Zhang W, Sargent DJ, Mandrekar S (2006). An adaptive dose-finding design incorporating both toxicity and efficacy. Stat Med.

[14] Wages NA, Tait C (2015). Seamless Phase I/II Adaptive Design for Oncology Trials of Molecularly Targeted Agents,. J Biopharm Stat.

[15] Wang M, Day R (2010). Adaptive Bayesian design for phase I dose-finding trials using a joint model of response and toxicity,. J Biopharm Stat.

[16] Thall P, Cook J (2004). Dose-Finding Based on Efficacy-Toxicity Trade-Offs. Biometrics.

[17] Anderlini P, Wu J, Gersten I, Ewell M, Tolar J, Antin JH, Adams R, Arai S, Eames G, Horwitz ME, McCarty J, Nakamura R, Pulsipher MA, Rowley S, Leifer E, Carter SL, DiFronzo NL, Horowitz MM, Confer D, Deeg HJ, Eapen M (2015). Cyclophosphamide conditioning in patients with severe aplastic anaemia given unrelated marrow transplantation: a phase 1–2 dose de-escalation study. Lancet Haematol.

[18] Shah N, Thall PF, Fox PS, Bashir Q, Shah JJ, Parmar S, Lin P, Kebriaei P, Nieto Y, Popat UR, Hosing CM, Cornelison A, Shpall EJ, Orlowski RZ, Champlin RE, Qazilbash MH (2015). Phase I/II trial of lenalidomide and high dose melphalan with autologous stem cell transplantation for relapsed myeloma. Leukemia.

[19] Chen TL, Estey EH, Othus M, Gardner KM, Markle LJ, Walter RB (2013). Cyclosporine Modulation of Multidrug Resistance in Combination with Pravastatin, Mitoxantrone, and Etoposide for Adult Patients with Relapsed/Refractory Acute Myeloid Leukemia (AML): A Phase 1/2 Study. Leuk Lymphoma.

[20] Briasoulis E, Aravantinos G, Kouvatseas G, Pappas P, Biziota E, Sainis I, Makatsoris T, Varthalitis I, Xanthakis I, Vassias A, Klouvas G, Boukovinas I, Fountzilas G, Syrigos KN, Kalofonos H, Samantas E (2013). Dose selection trial of metronomic oral vinorelbine monotherapy in patients with metastatic cancer: a hellenic cooperative oncology group clinical translational study. BMC Cancer.

[21] Thall PF (2010). Bayesian Models and Decision Algorithms for Complex Early Phase Clinical Trials. Stat Sci.

[22] Thall P, Herrick R, Nguyen H, Venier J, Norris J (2014). Effective sample size for computing prior hyperparameters in Bayesian phase I-II dose-finding. Clin Trials.

[23] Thall P, Cook J, Estey E (2006). Adaptive dose selection using efficacy-toxicity trade-offs: illustrations and practical considerations,. J Biopharm Stat.

[24] Cook JD. Efficacy-Toxicity trade-offs based on L-p norms: Technical Report UTMDABTR-003-06. Technical report. 2006.

[25] Yuan Y, Nguyen HQ, Thall PF (2016). Bayesian Designs for Phase I–II Clinical Trials.

[26] Jin IH, Liu S, Thall PF, Yuan Y (2014). Using Data Augmentation to Facilitate Conduct of Phase I-II Clinical Trials with Delayed Outcomes. J Am Stat Assoc.

[27] Wadhwa J, Szydlo RM, Apperley JF, Chase A, Bua M, Marin D, Kanfer E, Goldman JM, Olavarria E. Factors affecting duration of survival after onset of blastic transformation of chronic myeloid leukemia Factors affecting duration of survival after onset of blastic transformation of chronic myeloid leukemia. 2012; 99(7):2304–9. doi:10.1182/blood.V99.7.2304. 10.1182/blood.v99.7.2304 11895760

[28] Cortes JE, Kim DW, Pinilla-Ibarz J, le Coutre P, Paquette R, Chuah C, Nicolini FE, Apperley JF, Khoury HJ, Talpaz M, DiPersio J, DeAngelo DJ, Abruzzese E, Rea D, Baccarani M, Müller MC, Gambacorti-Passerini C, Wong S, Lustgarten S, Rivera VM, Clackson T, Turner CD, Haluska FG, Guilhot F, Deininger MW, Hochhaus A, Hughes T, Goldman JM, Shah NP, Kantarjian H (2013). A phase 2 trial of ponatinib in Philadelphia chromosome-positive leukemias. N Engl J Med.

[29] R Core Team: R: A Language and Environment for Statistical Computing. Vienna: R Foundation for Statistical Computing; 2014. http://www.R-project.org/.

[30] Soetaert K, Herman PMJ (2009). A Practical Guide to Ecological Modelling. Using R as a Simulation Platform.

[31] Yap C, Billingham L, Craddock C, O’Quigley J (2015). Dose transition pathways: a design, analysis and operational tool for dose-finding trials using model-based designs. Trials.

[32] Thall PF, Nguyen HQ, Zinner RG (2017). Parametric dose standardization for optimizing two-agent combinations in a phase I-II trial with ordinal outcomes. J R Stat Soc Series C: Appl Stat.

[33] Monahan J, Genz A (1997). Spherical-Radial Integration Rules for Bayesian Computation. J Am Stat Assoc.

[34] Brock K. clintrials v0.1.4. GitHub, Zenodo. 2016. doi:10.5281/zenodo.164621, https://zenodo.org/record/164621, https://github.com/brockk/clintrials.

[35] Le Tourneau C, Lee JJ, Siu LL (2009). Dose escalation methods in phase i cancer clinical trials. J Natl Cancer Inst.

